# Bone scintigraphy after osteochondral autograft transplantation in the knee: 13 patients followed for 4 years

**DOI:** 10.3109/17453671003587101

**Published:** 2010-04-06

**Authors:** Niels B Kock, Esther van Tankeren, Wim J G Oyen, Ate B Wymenga, Job L C van Susante

**Affiliations:** ^1^Department of Orthopaedics, Sint Maartenskliniek, Nijmegenthe Netherlands; ^2^Department of Nuclear Medicine, Radboud University Nijmegen Medical Centre, Nijmegenthe Netherlands; ^3^Department of Orthopaedics, Rijnstate Hospital, Arnhemthe Netherlands

## Abstract

**Background and purpose** Autologous osteochondral transplantation (OCT) is an established method of treating articular cartilage defects in the knee. However, the potential for donor site morbidity remains a concern. Both the restoration of the original cartilage defect and the evolution of the donor site defects can be evaluated by bone scintigraphy. Thus, we performed a prospective bone scintigraphic evaluation in patients who were treated with OCT.

**Patients and methods** In 13 patients with a symptomatic articular cartilage defect, bone scintigraphies were obtained preoperatively, 1 year after osteochondral transplantation, and finally at an average follow-up of 4 (2.5–5.5) years. The evolution of scintigraphic activity was evaluated for both the recipient and the donor site. Parallel, clinical scoring was performed using the Lysholm knee scoring scale, the Cincinnati knee rating system, and the Tegner activity score.

**Results** The bone scintigraphic uptake was elevated at the involved femoral condyle preoperatively, and gradually decreased to normal levels in 7 of 11 cases. The originally normal uptake at the trochlea increased 1 year after transplantation. Then, a gradual decrease in uptake occurred again at this donor site to remain elevated at the final scintigraphy. A correlation was found between elevated scintigraphic activity and the presence of retropatellar crepitus. The mean Lysholm and Cincinnati scores had increased 1 year after transplantation. The mean Tegner score had increased 3 years after transplantation.

**Interpretation** Elevated bone scintigraphic activity from an osteochondral lesion in the knee can be restored with OCT. However, increased scintigraphic activity is introduced at the donor site, which becomes reduced with longer follow-up. The use of fairly large osteochondral plugs appears to correlate with retropatellar crepitus and increased scintigraphic activity, and is not therefore recommended.

Focal cartilage damage in the knee is most commonly caused by trauma and osteochondritis dissecans. The choice of treatment of such a cartilage lesion depends mainly on the size and location of the lesion, and the age of the patient. In osteochondritis dissecans lesions, no treatment is necessary in children with a stable lesion. Surgical treatment is usually advocated for unstable lesions, in case of persisting symptoms, or if skeletal maturity is imminent. Surgery usually consists of removal of a loose body and abrasion and drilling of the defect or fixation of the loose body with bone, or metallic or bio-absorbable pins. Over the past decade, autologous chondrocyte implantation and osteochondral autografting have also been used (Farnworth 2000, Cain and Clancy 2001).

Clinical outcome after osteochondral transplantation has shown promising results (Jakob et al. 2002, Bentley et al. 2003, Hangody and Fules 2003, Horas et al. 2003). Nevertheless, there still remain matters of concern with this technique, such as survival of the transplanted hyaline cartilage and potential donor site morbidity. The donor site defect at the trochlear area appears to heal with fibrocartilage; this area is subject to contact pressure and it is uncertain whether this may lead to degenerative changes and symptoms (Simonian et al. 1998, Garretson, III et al. 2004).

Bone scintigraphy reveals increased osteoblastic activity, which indicates degenerative changes or bone repair. We have not found any previous studies on bone scintigraphy following osteochondral transplantations in the knee. One would expect increased scintigrafic activity at the site of the original articular cartilage lesion. Whether this increased activity can be restored with osteochondral transplantation and whether the donor defect may lead to an increased activity is unclear.

We evaluated 13 patients who were treated with autologous osteochondral transplantation for a focal osteochondral knee lesion, with repeated bone scintigraphies of the donor and the recipient site up to 5 years after surgery.

## Patients and methods

During 3 years, 13 patients (mean age 33 (23–48) years, 8 men) were operated for isolated full-thickness cartilage lesions of the femoral condyle either caused by trauma (7 cases) or osteochondritis dissecans (6 cases). The medial condyle was affected in 10 patients, and the lateral condyle in 3 patients. The average follow-up was 49 (31–65) months.

All patients had had at least 1 earlier surgical intervention on the knee. All had a prior arthroscopy for debridement of the defect; in addition, there were 2 prior ACL reconstructions, 1 MCL stabilization, 1 partial meniscectomy, 1 tibial osteotomy, and 1 tuberositas transposition. All knees had a neutral to slightly valgus alignment and no signs of osteoarthritis were present except for 1 patient who—besides the osteochondral lesion in the medial compartment—had arthrosis in the lateral compartment. The indication for osteochondral transplantation of this patient was questionable. Conversion to a total knee arthroplasty was performed 3 years after the osteochondral transplantation. Approval from a local ethical committee was obtained and informed consent was given by all patients.

### Surgery

All osteochondral transplantations were performed by the same orthopedic surgeon (ABW). A medial arthrotomy of the knee was performed and the articular defect was debrided. The size of the defect was then determined and reconstruction of the articular surface with osteochondral plugs was planned. Cylindrical subchondral plugs (mean 2.4 (1–4) plugs) were taken from the cartilage defect perpendicular to the surface (recipient site). Matching osteochondral cylinders were harvested from the lateral trochlear region (donor site) using a cylindrical diamond cutter (Synthes, Zeist, the Netherlands). The diameter of the plug overshot the diameter of the recipient site by 1 mm for press-fit. The diameter of the plugs varied from 7.5 to 12.9 mm (Table 1). The plug was tampered into the defect until the cartilage cap of the plug reached the same level as the surrounding cartilage. The same approach was used if more plugs were needed to fill the condylar defect. The donor site defects were filled with press-fitted osteo-periosteal plugs harvested from the proximal tibia (van Susante et al. 2003, Kock et al. 2004). All patients had restricted weight bearing for 6 weeks.

**Table 1.** The plugs

Patient	Total no. of plugs	Plug diameter (no.)
1	1	12.9
2	2	8.5
3	2	7.5 / 8.5
4	4	7.5 (2) / 9.6 (2)
5	4	8.5 (1) / 11.7 (3)
6	1	11.7
7	4	7.5 (1) / 8.5 (3)
8	1	9.6
9	1	7.5
10	3	7.5
11	2	8.5
12	3	9.6 (1) / 11.7 (2)
13	3	9.6 (3)

### Clinical evaluation

The Lysholm knee scoring scale, the Cincinnati knee rating system, and the Tegner activity score were used to evaluate clinical outcome (Lysholm and Gillquist 1982, Noyes et al. 1983, Tegner and Lysholm 1985). In addition, the presence of patello-femoral crepitus was separately scored as none, moderate, with mild pain, and with more than mild pain.

All patients were evaluated preoperatively, after 1 year, and at the final follow-up.

### Bone scintigraphy

At the clinical evaluations, bone scintigraphy was also performed. Bone scintigraphies were obtained 5 min and 3 h after administering 600 MBq Tc-99m-MDP intravenously. They were evaluated by two nuclear physicians. Both the recipient and the donor site were evaluated at each time interval. Uptake was visually compared to that in normal, surrounding bone and scored as follows: 0 = normal, equal to surrounding bone, 1 = slightly elevated, 2 = moderate (clearly discernable from surrounding bone), and 3 = strongly elevated. Of the 35 bone scintigraphies that were initially scored differently by the observers, consensus could be obtained in only two.

### Statistics

All scores were grouped according to the time of testing: preoperatively, 1 year, and 3 years postoperatively. For evaluation of differences in the Lysholm and Cincinnati scores, the general linear repeated-measures model was used. The 3 follow-up points were entered as a within-subject factor with 3 levels (preoperatively, 1 year, and 3 years postoperatively). The Lysholm and Cincinnati scores were inserted into the model as dependent variables. When a statistically significant effect of follow-up moment was found, a post-hoc test (Bonferroni) was performed to identify which follow-up moments were significantly different. For comparison of differences in Tegner score, presence of retropatellar crepitus, and scintigraphic activity for the 3 follow-up moments, the non-parametric Wilcoxon signed ranks test was used. For testing of correlation, the non-parametric Spearman’s correlation test was used. For all tests, p-values less than 0.05 were considered to be statistically significant. For all statistical analyses, SPSS 14.0 for Windows was used.

## Results

### Clinical evaluation

All 3 clinical knee-scoring systems showed moderate improvement (Table 2). Retropatellar crepitus was found in 3 of the 13 patients preoperatively and 1 of these patients had mild pain. 1 year after surgery 10 patients had retropatellar crepitus, 2 with mild pain and 1 with severe pain. At the latest follow-up, only 1 patient did not experience retropatellar crepitus and again 2 experienced mild pain and 1 experienced severe pain. The presence of retropatellar crepitus with or without pain increased after surgery, both at 1 year (p = 0.008) and at 3 years postoperatively (p = 0.005). No statistically significant differences were found between the results at 1 year and 3 years after surgery.

**Table 2.** Clinical scores

	Preoperatively	Postoperatively: 1 year	Postoperatively: latest follow-up	p-value ^a^		
				0–1	1–4	0–4
Lysholm	64 (40–81)	79 (48–95)	77 (40–100)	0.01	1.0	0.05
Cincinnati	67 (54–79)	76 (57–93)	78 (48–97)	0.01	1.0	0.09
Tegner	2.4 (1–3)	3.4 (1–9)	3.4 (1–7)	0.06	0.8	0.03
**^a^** P-values for the difference in clinical scores are indicted between the respective years of follow-up (preoperatively vs. 1, 1 vs. 4 and preoperatively vs. 4 years of follow-up).					

No correlations were found between plug sizes and numbers on the one hand and clinical outcome or retropatellar crepitus on the other.

### Bone scintigraphy

All patients were scanned preoperatively and at 1 year postoperatively. At the third follow-up, bone scintigraphy was done in 11 of 13 the patients. The last bone scintigraphy was not performed in the patient who had had a total knee arthroplasty and in another patient who was not willing to return. The final bone scintigraphy was performed in 3 patients after 3 years, in 3 other patients after 4 years, and in 5 patients after 5 years.

At the cartilage defect (recipient site), only 2 patients had normal activity at the preoperative scintigraphy. 7 patients showed slightly elevated activity levels, 3 showed moderately elevated activity levels, and 1 showed strongly elevated activity levels on this scan. At 1 year after surgery, 10 patients showed the same or slightly increased activity at the recipient site in comparison to the preoperative levels. At the final scintigraphy, only 3 patients still had slightly elevated activity levels and 1 patient had moderately elevated activity levels. In the remaining 11 patients, the originally elevated activity at the cartilage defect had been restored to normal. These changes in scintigraphic activity approached statistical significance when compared preoperatively and at final follow-up (p = 0.07), and they were statistically significant when compared at 1 year and at final follow-up (p = 0.02).

At the lateral trochlear areas, later to be used as donor site for osteochondral plug harvesting, all scans demonstrated normal activity levels preoperatively. 1 year after the transplantation, the scintigraphy revealed increased activity at the trochlear groove in 9 patients whereas in 4 patients the activity remained normal. In the 9 patients with elevated activity, it was slightly elevated in 5 patients, moderately elevated in 3 patients, and strongly elevated in 1 (Figure). At final follow-up, in 3 patients activity increased from zero to mild levels at the donor sites, in 3 patients activity remained unchanged, and in 5 patients activity levels had decreased. Eventually, only 2 patients showed normal activity levels at the final bone scintigraphy and 9 patients appeared to have progressed from normal preoperatively to slightly elevated levels at the latest follow-up. The difference between preoperative and postoperative activity levels was significant both at the 1-year follow-up (p = 0.006) and at the final follow-up (p = 0.005).

**Figure:**
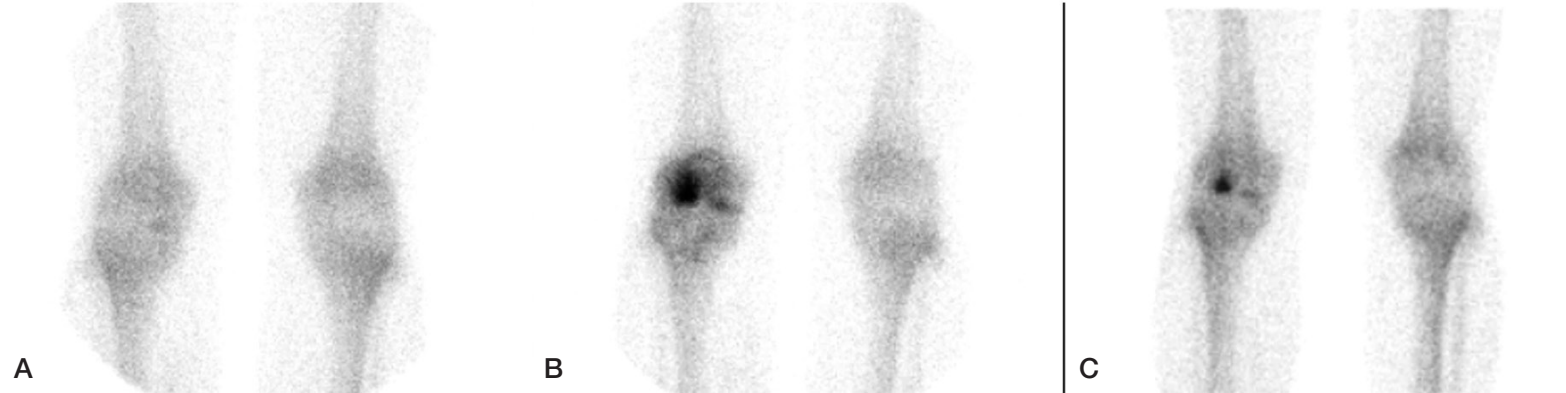
Scintigraphy in patient number 7 (see Table 1). A. Preoperatively: mild activity at the medial condyle. B. 1 year postoperatively: strongly elevated activity at the lateral trochlea and moderate activity at the medial condyle. C. 3 years postoperatively: reduced activity at the medial condyle and persistant activity at the lateral trochlea.

A correlation was found between the presence of retropatellar crepitus and elevated bone scintigraphic activity at the trochlear area (p = 0.007). No correlation was found between plug sizes and numbers on the one hand and scintigraphic activity on the other.

## Discussion

The clinical scoring did not improve as much as we had anticipated, and better clinical results have been reported (Jakob et al. 2002, Bentley et al. 2003, Hangody and Fules 2003, Chow et al. 2004, Karataglis et al. 2006). In our patients, donor site morbidity was a problem with retropatellar crepitus and pain, and increased scintigraphic activity. Donor site morbidity has been reported (Jakob et al. 2002, Bentley et al. 2003, Hangody and Fules 2003, van Susante et al. 2003, Alford and Cole 2005, Marcacci et al. 2007) but there is no consensus as whether donor site problems are of clinical relevance.

The presence of retropatellar crepitus and the limited improvement in clinical scoring in our patients may be explained by the fact that most patients had concomitant knee problems and rather large osteochondral donor plugs were used, with diameters up to 13 mm. The number of harvested plugs and their diameter are factors that may influence the clinical outcome (Jakob et al. 2002, Hangody and Fules 2003).

We harvested plugs from the lateral trochlear area and one could argue whether less weight bearing may be present at the medial trochlear area. We still chose the lateral trochlea as donor site because of its larger surface area, and we believe that the diameter and number of plugs used are more related to crepitus than to whether the lateral or medial trochlea is used as donor site.

We used an osteoperiosteal plug from the proximal tibia to fill the donor site defects. Periosteal tissue is known to give rise to hypertrophy and crepitus when used for autologous chondrocyte implantation (Niemeyer et al. 2008), and one could question whether this periosteum may have contributed to the high rates of crepitus. We do not believe that this is likely since in an earlier animal experiment we found that these osteoperiosteal plugs commonly failed to stimulate repair of the donor site due to osteoclastic resorption (van Susante et al. 2003). Thus, we feel that these additional grafts were not of any importance and we have abandoned this technique.

### Scintigraphy – recipient site

We found elevated uptake on bone scintigraphy at the site of the cartilage defect in 11 of 13 patients on presentation. This increased uptake approached normal activity levels, in most cases following osteochondral transplantation. This finding makes us believe that adequate joint resurfacing can be achieved and progressive degeneration of the femoral condyle can be reversed by means of osteochondral transplantation.

In addition, the normalized uptake on bone scintigraphy at the recipient site after an average of 4 years indicates adequate graft incorporation in the adjacent subchondral bone. In the absence of adequate incorporation of the graft or with subchondral cyst formation, elevated uptake would have persisted. Incorporation of the graft in the subchondral bone is supported by a retrieval case report on a patient who had had a knee arthroplasty after clinical failure following the osteochondral transplantation. Histology revealed incorporated grafts with vital hyaline cartilage caps (Kock et al. 2004).

2 of 13 patients did not have elevated scintigraphic activity at the lesion site on presentation. The reason is obscure, and surprisingly these 2 patients had a poor clinical result. This finding raises the question of whether the cartilage lesions were indeed symptomatic, and more importantly whether bone scintigraphy may play a role in selection of patients who are suitable for osteochondral transplantation.

### Scintigraphy – donor site

Preoperatively, scintigraphies were normal at the trochlear level. 1 year after the transplantation, nearly all patients had developed a markedly increased activity at the donor site. At the time of the last scintigraphy, however, after an average time of 4 years, the activity had decreased. There was a positive correlation between activity levels and retropatellar crepitus with or without pain.

Earlier publications have described the rather benign clinical course of this crepitus, with gradually declining symptomatology (Jakob et al. 2002, Hangody and Fules 2003). These findings are supported by our scintigraphic data.

In summary, bone scintigraphy appears to be a good diagnostic tool for following osteochondral transplantations, especially for distinction between recipient and donor site morbidity.
